# Associated factors of smoking behaviors among industrial workers in Myanmar: the role of modifying factors and individual beliefs, guided by the health belief model

**DOI:** 10.3389/fpubh.2025.1655922

**Published:** 2025-09-16

**Authors:** Myo Zin Oo, Soe Sandi Tint, Alessio Panza, Sathirakorn Pongpanich, Pramon Viwattanakulvanid, Somdeth Bodhisane, Amaraporn Rerkasem, Kittipan Rerkasem

**Affiliations:** ^1^Environmental - Occupational Health Sciences and NonCommunicable Diseases Research Center, Research Institute for Health Sciences, Chiang Mai University, Chiang Mai, Thailand; ^2^Global Health and Chronic Conditions Research Center, Chiang Mai University, Chiang Mai, Thailand; ^3^Department of Family Medicine, Faculty of Medicine, Chiang Mai University, Chiang Mai, Thailand; ^4^College of Public Health Sciences, Chulalongkorn University, Bangkok, Thailand; ^5^Research Center for Infectious Disease and Substance Use, Research Institute for Health Sciences, Chiang Mai University, Chiang Mai, Thailand; ^6^Clinical Surgical Research Center, Department of Surgery, Faculty of Medicine, Chiang Mai University, Chiang Mai, Thailand

**Keywords:** health belief model, health beliefs, health knowledge, industrial workers, Myanmar, occupational health, self-efficacy, smoking behaviors

## Abstract

**Introduction:**

Smoking is a preventable behavioral risk factor for both communicable and noncommunicable diseases, with particularly strong impacts on noncommunicable diseases. We aimed to examine the associations between modifying factors, individual beliefs, and smoking behaviors, including quit attempts and smoking intensity, among industrial workers in Myanmar.

**Methods:**

Our cross-sectional study utilized baseline data collected in 2018 from a longitudinal quasi-experimental study involving 292 male industrial workers in Mandalay, Myanmar. Employing the Health Belief Model, we examined the associations of modifying factors (age, sex, marital status, education, income, smoking initiation age, duration, quit intention, and health knowledge) and individual beliefs (perceived susceptibility, severity, barriers, benefits, and self-efficacy) with smoking behaviors, specifically quit attempts and smoking intensity. Data were collected via structured interviews and analyzed using descriptive statistics and multivariable logistic regression models adjusted for potential confounders, with significance set at *p* < 0.05.

**Results:**

The median age of participants was 28 years, with 90.4% not having attempted to quit smoking and 47.6% identified as high-intensity smokers. Health knowledge was significantly associated with lower odds of being a high-intensity smoker in both crude (OR = 0.65, 95% CI: 0.49, 0.88, *p* = 0.005) and adjusted models (AOR = 0.53, 95% CI: 0.38, 0.75, *p* < 0.001). Higher self-efficacy also significantly reduced the odds of being a high-intensity smoker in the adjusted model (AOR = 0.93, 95% CI: 0.86, 0.99, *p* = 0.044).

**Conclusion:**

Our study reveals complex interactions between modifying factors and individual beliefs associated with smoking behaviors among industrial workers in Myanmar. The need for tailored health education interventions for industrial workers to enhance health knowledge and self-efficacy.

## 1 Introduction

Tobacco smoking is a leading preventable cause of death globally (1), claiming over eight million lives each year, with an increasing burden particularly in low- and middle-income countries (LMICs) (2). In Myanmar, an LMIC, smoking prevalence remains high, especially among men (36.5% in 2022) (3), significantly contributing to the country’s burden of both communicable and noncommunicable diseases (NCDs), including cardiovascular conditions, cancers, and chronic respiratory illnesses, and infectious diseases such as tuberculosis (4). Vascular diseases such as coronary artery disease (5), stroke (6), and peripheral artery disease (7) are notably vulnerable to smoking-related harm. Industrial workers represent a high-risk group for tobacco use due to occupational environments characterized by physical and psychosocial stressors (8, 9), peer influence (10), and limited access to health promotion and cessation programs (11). This increased prevalence heightens their risk for lung diseases and cardiovascular issues (12), exacerbating the overall NCD burden, while also leading to productivity losses, workplace accidents, and rising healthcare costs.

Although behavioral models like the Theory of Planned Behavior (TPB) (13), the Transtheoretical Model (TTM) (14, 15), and Social Cognitive Theory (SCT) (16) have been employed to understand health behaviors, the Health Belief Model (HBM) (17–19) remains one of the most widely used in health education (HE), disease prevention, and community interventions. The HBM’s focus on health motivation and preventive behavior makes it particularly suitable for exploring smoking behaviors among at-risk populations such as industrial workers (20). It comprises six key constructs (18): perceived susceptibility, perceived severity, perceived barriers, perceived benefits, self-efficacy, and cues to action. Definitions of these constructs are provided in Supplementary material 1 (18). However, research applying the HBM in Myanmar’s industrial settings is scarce. Addressing this gap is essential for developing targeted interventions in occupational contexts where health promotion resources are often limited.

**Figure 1:**
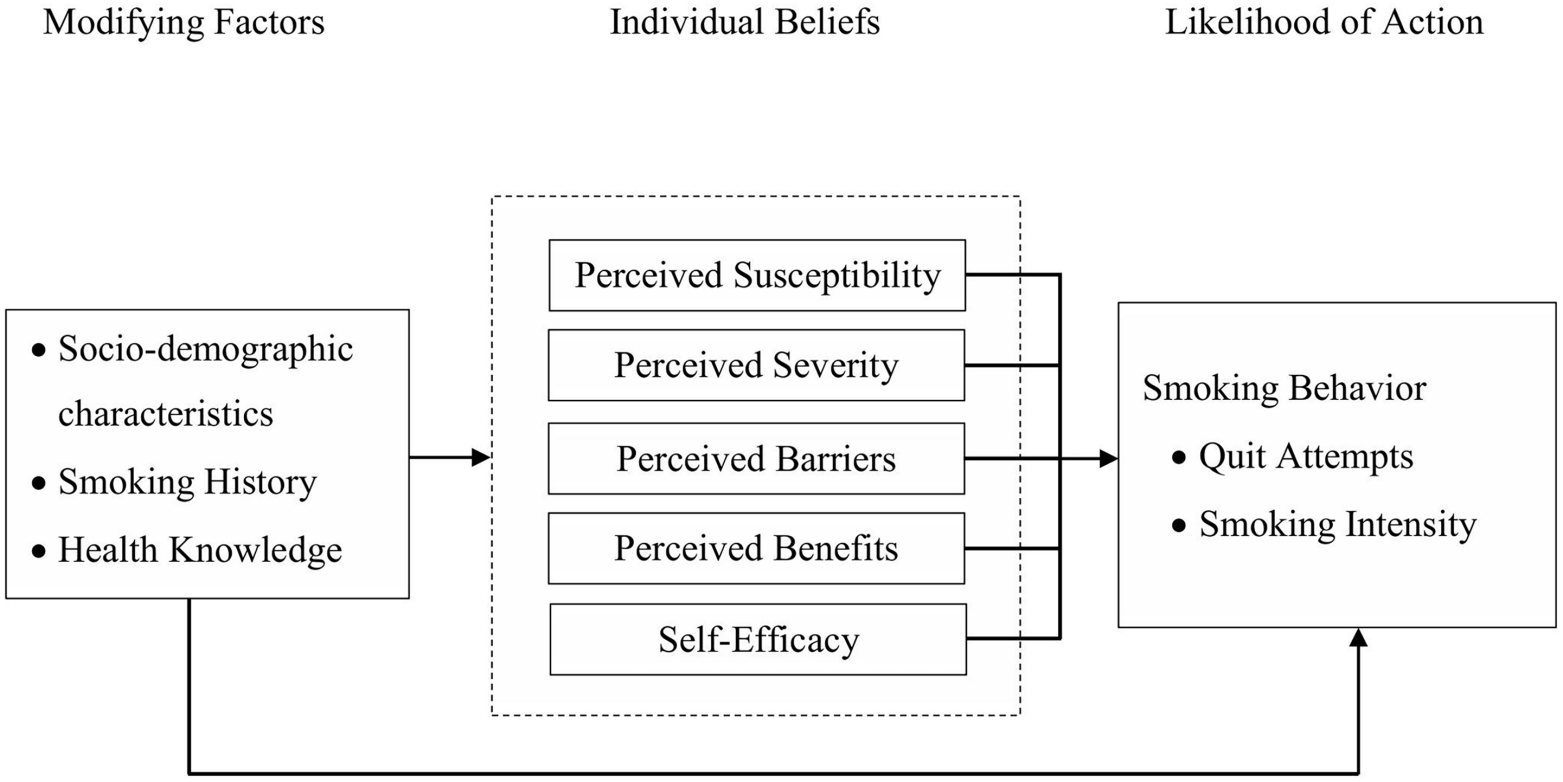
Adapted health belief model theoretical framework for the study.

The adapted conceptual framework guiding our study (Figure 1) categorizes predictors into two domains: modifying factors and individual beliefs, reflecting their theorized roles in shaping the likelihood of taking health-related action. While the framework outlines potential intermediary pathways, our hypotheses focused on the direct effects of these domains on smoking behavior outcomes. Additionally, quit smoking intention, though not a core HBM construct, was included as a motivational modifying factor due to its demonstrated role in behavior change (21). Thus, our study aimed to examine the associations between modifying factors, individual beliefs, and smoking behaviors, including quit attempts and smoking intensity, among industrial workers in Myanmar.

## 2 Methods

### 2.1 Study design

We employed a cross-sectional analytical design using the baseline dataset from our longitudinal quasi-experimental study (17), which involved intervention and control groups among industrial workers in Mandalay, Myanmar. For this analysis, data from both groups were combined and treated as a single cross-sectional dataset collected prior to any intervention exposure. This standalone analysis addresses predictors of smoking behavior distinct from the intervention outcomes.

### 2.2 Study setting, study population, and study period

Our study was conducted among industrial workers in the Mandalay Industrial Zone (MIZ), located in Mandalay region, which is Myanmar’s second-largest city and a key economic and manufacturing hub (22). The MIZ was purposively selected based on its high density of manufacturing factories and large industrial workforce (23), which provided a relevant and accessible population for research on smoking behaviors in occupational settings.

Participants were recruited from selected industries that met predefined eligibility criteria, including industry size, type of manufacturing, salary range, gender distribution, and geographic location. The study focused on workers currently employed in these industries. Our study’s baseline data were collected in June 2018.

### 2.3 Inclusion and exclusion criteria

Eligible participants included industrial workers aged 18 years or older, who current smoked at least one cigarette per day, worked full-time in selected industries in the MIZ, were literate in Burmese, and consented voluntarily. Individuals were excluded if they had current or prior enrollment in formal smoking cessation programs, suffered from illnesses or cognitive impairments that could interfere with participation, or were pregnant. No female workers could be identified as eligible participants during recruitment, largely attributable to gender norms and employment patterns within industrial settings in Myanmar (17). Specifically, smoking is highly stigmatized among women in this context, and female employees were either not present in eligible roles or did not self-identify as smokers. Consequently, the study included only male industrial workers.

These criteria were applied during baseline data collection, which preceded any intervention exposure and forms the basis for the present cross-sectional analysis.

### 2.4 Sample size determination

Our present analysis included 292 male industrial workers, based on baseline (pre-intervention) data collected from our longitudinal quasi-experimental study (17), comprising both intervention (*n* = 146) and control (*n* = 146) groups. As this is a standalone cross-sectional analysis using baseline data only, no additional *a priori* sample size calculation was performed. However, the existing sample is considered appropriate for several reasons. First, it meets the commonly recommended threshold of 20 observations per predictor variable in multivariable regression, which supports model stability and reliability (14 predictors × 20 = 280; *N* = 292) (24). This exceeds traditional rules such as the “10-per-predictor” criterion, which recent literature (25) has noted may be insufficient in some contexts. Second, this analysis focuses on identifying associations rather than causal effects, allowing for more flexibility in statistical power requirements. Finally, the original sample size (17) was determined through standard power analysis for comparing two independent proportions, using a 0.05 significance level, 80% power, and an anticipated 30% dropout rate. Collectively, these considerations support the adequacy of the current sample for our planned analysis.

### 2.5 Sampling technique

A multistage sampling technique was employed. From the initial list of 794 registered industries in the MIZ, 17 met the predefined eligibility criteria (17). Of these, two industries were randomly selected using a computer-generated randomization process. In each selected industry, participants were recruited through systematic random sampling after being informed about the study and providing written informed consent. Recruitment continued until 292 male smokers (146 per industry) were enrolled. As data were collected prior to any intervention, this analysis reflects a cross-sectional snapshot of the study population and is analytically distinct from the longitudinal design of the original quasi-experimental study.

### 2.6 Data collection tools and process

Data were collected through interviewer-administered face-to-face interviews using a structured questionnaire. The instrument used in this study was the same tool developed and validated for our earlier quasi-experimental study (17), adapted from established sources in the literature (20, 26–32) and grounded in the theoretical constructs of the HBM to ensure construct validity. The questionnaire captured predictor variables: modifying factors and individual beliefs, as well as outcome variables related to smoking behaviors (likelihood of action), such as quit attempts and smoking intensity, with the latter measured by the number of cigarettes smoked per week. The questionnaire was reviewed by three experts in tobacco-related research for content validity. To ensure linguistic accuracy, the questionnaire underwent a back-translation process. It was first translated from English to Burmese, and then translated back from Burmese to English to ensure consistency and clarity.

### 2.7 Predictor variables

#### 2.7.1 Modifying factors

This section included variables such as age, sex, marital status, education level, monthly income, age at smoking initiation, duration of smoking (in years), and quit smoking intention (yes or no). Additionally, health knowledge was included as a modifying factor. It was assessed using 10 items designed to evaluate participants’ knowledge of smoking-related diseases, adapted from a previous study (26) and aligned with more recent research (27, 28). Responses were categorized as “unknown,” “not sure,” “incorrect,” or “correct.” Each “correct” response was assigned a score of one, while the others were assigned a score of zero. A total score was calculated and summarized using the median and interquartile range (IQR).

#### 2.7.2 Individual beliefs

This section assessed four key perceptions based on the HBM, using a 5-point Likert scale adapted from previous studies (29, 30) and aligned with more recent research (20). The constructs measured included perceived susceptibility (11 positive statements), perceived severity (10 positive statements), perceived barriers (7 negative statements), and perceived benefits (5 positive statements). The scale ranged from “strongly disagree” to “strongly agree.” The total scores were calculated and summarized using the median and IQR.

Moreover, self-efficacy was included under individual beliefs. It was assessed using the six-item Smoking Abstinence Self-Efficacy Questionnaire (SASEQ) developed by Spek et al. (31), which measures participants’ confidence in resisting smoking in various situations. The use of this tool is supported by recent studies (32). The scale ranged from “certainly not” (0) to “certainly” (4). A total score was calculated and summarized using the median and IQR.

Detailed item-level operationalization for all these constructs is provided in Supplementary material 1.

### 2.8 Outcome variables

#### 2.8.1 Smoking behavior

##### 2.8.1.1 Quit attempts

Participants were asked whether they had attempted to quit smoking in the past 3 months. Responses were categorized as a binary variable: 0 = no quit attempt, and 1 = at least one quit attempt.

##### 2.8.1.2 Smoking intensity

Participants reported their average weekly cigarette consumption. This continuous variable was dichotomized at the sample median (23 cigarettes/week, approximately 3.3 per day). Although the most recent national survey on Diabetes Mellitus and NCD risk factors in Myanmar (2014) (33) reported a lower mean of 1.9 cigarettes per day among men, the higher median observed in our sample justifies using this cutoff to classify low-intensity smokers (≤ 23/week) and high-intensity smokers (> 23/week).

Data collection was conducted by three trained interviewers with prior experience in health surveys. They underwent additional training in ethical conduct, building trust with participants, and ensuring standardized administration. Interviews were conducted at the respective industrial sites.

### 2.9 Data analysis

We conducted all analyses in SPSS version 25. Descriptive statistics were applied to summarize participants’ modifying factors (age, sex, marital status, education level, monthly income, age at smoking initiation, duration of smoking, quit smoking intention, and health knowledge), individual beliefs (perceived susceptibility, severity, barriers, benefits, and self-efficacy), and smoking behavior outcomes. Frequencies and percentages were reported for categorical data, while medians and IQRs summarized continuous data.

Prior to multivariable analysis, bivariate analyses were conducted to examine associations between each variable and the two binary smoking behavior outcomes: quit attempts and smoking intensity. Monthly income was treated as a continuous variable and rescaled per 10,000 MMK to enhance the interpretability. To address multiple comparisons, false discovery rate (FDR) adjustment using the Benjamini-Hochberg procedure was applied to the bivariable *p*-values. Multicollinearity was assessed using variance inflation factors (VIFs). Most variables showed VIFs well below 5, indicating low multicollinearity. Age and duration of smoking had very high VIFs (41.7 and 35.3, respectively), exceeding the conservative threshold of 5, and were excluded from multivariable analysis due to their non-significant bivariate associations (*p* ≥ 0.25). Age at smoking initiation had a VIF of 5.4, slightly above 5, but was retained due to its theoretical relevance, a borderline *p*-value (0.118) in bivariate analysis, and robustness in sensitivity analyses for smoking intensity. Variables with a crude bivariable *p*-value below 0.25 were selected for inclusion in the multivariable model. This threshold is commonly used in epidemiological research to avoid prematurely excluding potentially important variables, allowing factors that may not reach conventional significance in bivariable tests but could be relevant in the multivariable context to be evaluated (34). Adjusted odds ratios (AORs) and 95% confidence intervals (CIs) were reported, with a significance level established at *p* < 0.05. As this analysis is based solely on baseline (pre-intervention) data, no longitudinal or group-based comparisons were conducted.

## 3 Results

Table 1 presents the participant characteristics (modifying factors) of the HBM constructs. All respondents were males (*N* = 292) with a median age of 28.00 years. More than half were married (54.1%), and the majority (78.1%) had completed high school or higher education. The median monthly income was 18.00 per 10,000 Myanmar Kyats (MMK), equivalent to approximately 180,000 MMK (around 86 USD; based on the Central Bank of Myanmar exchange rate of 1 USD = 2,100 MMK on January 30, 2025) (35). Respondents had a median smoking initiation age of 18.00 and a duration of 10.00 years. Over half of the respondents (57.5%) reported no intention to quit smoking. The health knowledge score ranged from 0 to 3, with a median score of 2.00.

**Table 1:** Participant characteristics (modifying factors) (*N* = 292).

Participant characteristics (modifying factors)	Number (*N*)	Percentage (%)
Age		
Median (IQR)	28.00 (24.00–35.00)	
Range (Min-Max)	18–60	
Sex (Male)	292	100.0
Marital status		
Single-never married	134	45.9
Married	158	54.1
Education level		
Primary school	27	9.2
Middle school	37	12.7
High school	113	38.7
University level	115	39.4
Monthly income (per 10,000 MMK)		
Median (IQR)	18.00 (18.00–20.00)	
Range (Min-Max)	14.00–25.00	
Age at smoking initiation		
Median (IQR)	18.00 (16.00–19.00)	
Range (Min-Max)	11–29	
Duration of smoking (in years)		
Median (IQR)	10.00 (7.00–17.75)	
Range (Min-Max)	1–42	
Quit smoking intention		
No	168	57.5
Yes	124	42.5
Health knowledge		
Median (IQR)	2.00 (1.00–2.00)	
Range (Min-Max)	0–3	

Table 2 displays individual beliefs based on HBM constructs. The median scores were 2.64 [41.0% of the Percentage of Maximum Possible Score (POMP)] for perceived susceptibility, 2.90 (47.5%) for perceived severity, 3.00 (50.0%) for perceived barriers, and 2.80 (45.0%) for perceived benefits, all measured on scale of 1–5. The median self-efficacy score was 7.00 (29.2% POMP) on a scale of 0–24.

**Table 2:** Individual beliefs based on HBM constructs (N = 292).

Individual beliefs	Theoretical scale range	Median (IQR)	Observed range (Min – Max)	POMP Score (%)^a^
Perceived susceptibility	1–5	2.64 (2.55–2.82)	2.09–3.09	41.0
Perceived severity	1–5	2.90 (2.70–3.00)	2.30–3.70	47.5
Perceived barrier	1–5	3.00 (2.86–3.14)	1.86–3.71	50.0
Perceived benefit	1–5	2.80 (2.40–3.20)	2.00–3.40	45.0
Self-efficacy	0–24	7.00 (5.00–13.00)	4.00–16.00	29.2

a Percentage of Maximum Possible Score (POMP) was calculated as: [(median - minimum possible score) / (maximum possible score - minimum possible score)] × 100.

Table 3 presents the smoking behavior outcomes (likelihood of action) related to the HBM constructs. The results indicate that a significant majority of participants (90.4%) have not attempted to quit smoking. Additionally, the respondents are nearly evenly split in terms of smoking intensity, with 52.4% classified as low-intensity smokers and 47.6% as high-intensity smokers.

**Table 3:** Smoking behavior outcomes (likelihood of action) (*N* = 292).

Smoking behavior outcomes	Number (*N*)	Percentage (%)
Quit attempts		
No attempt	264	90.4
At least one attempt	28	9.6
Smoking intensity		
Low-intensity smokers	153	52.4
High-intensity smokers	139	47.6

Table 4 interprets association between HBM constructs and smoking behavior outcomes. Higher self-efficacy was significantly associated with increased odds of quit attempts in the crude model (OR = 1.11, 95% CI: 1.01, 1.23, *p* = 0.029) but lost significance in the adjusted model (*p* = 0.163). For smoking intensity, higher health knowledge showed a significant reduction in the likelihood of being a high-intensity smoker in both the crude (OR = 0.65, 95% CI: 0.49, 0.88, *p* = 0.005) and adjusted models (AOR = 0.53, 95% CI: 0.38, 0.75, *p* < 0.001). Higher self-efficacy was also significantly associated with lower odds of being a high-intensity smoker in the adjusted model (AOR = 0.93, 95% CI: 0.86, 0.99, *p* = 0.044).

**Table 4:** Associations between HBM constructs and smoking behavior outcomes.

Variables	Quit attempts					Smoking intensity				
	Crude OR (95% CI)	*p*-value	FDR-adjusted *p*-value^a^	Adjusted OR (95% CI)^b^	*p*-value	Crude OR (95% CI)	*p*-value	FDR-adjusted *p*-value^a^	Adjusted OR (95% CI)^b^	*p*-value
Modifying factors										
Age	1.00 (0.96, 1.04)	0.981	0.981			0.99 (0.97, 1.02)	0.608	0.719	—	—
Marital status		0.130	0.439		0.223		0.450	0.650		—
Single-never married	1 (Ref)			1 (Ref)		1 (ref)			—	
Married	1.89 (0.83, 4.35)			1.76 (0.71, 4.38)		0.84 (0.53, 1.33)			—	
Education level		0.252	0.655		0.659		0.409	0.650		—
Primary school	1 (Ref)			1 (Ref)		1 (ref)			—	—
Middle school	2.92 (0.56, 15.32)	0.206		1.92 (0.34, 10.81)	0.458	0.58 (0.21, 1.61)	0.297		—	—
High school	1.21 (0.25, 5.89)	0.810		1.03 (0.19, 5.31)	0.974	1.14 (0.49, 2.63)	0.767		—	—
University level	1.06 (0.22, 5.22)	0.942		0.98 (0.19, 5.16)	0.979	0.99 (0.43, 2.28)	0.976		—	—
Monthly income (per 10,000 MMK)	0.86 (0.69, 1.05)	0.135	0.439	0.89 (0.71, 1.12)	0.331	1.03 (0.92, 1.16)	0.579	0.719	—	—
Age at smoking initiation	1.04 (0.93, 1.17)	0.485	0.981	—	—	0.94 (0.88, 1.02)	0.118	0.308	0.96 (0.89, 1.04)	0.336
Duration of smoking (in years)	0.99 (0.95, 1.04)	0.855	0.981	—	—	1.01 (0.98, 1.03)	0.671	0.727	—	—
Quit smoking intention	1.19 (0.55, 2.61)	0.656	0.981	—	—	1.48 (0.93, 2.36)	0.099	0.308	1.30 (0.80, 2.12)	0.288
Health knowledge	0.89 (0.56, 1.45)	0.661	0.981	—	—	0.65 (0.49, 0.88)	0.005*	0.065	0.53 (0.38, 0.75)	<0.001**
Individual beliefs										
Perceived susceptibility	1.31 (0.23, 7.52)	0.764	0.981	—	—	1.13 (0.40, 3.17)	0.817	0.817	—	—
Perceived severity	3.96 (0.96, 16.42)	0.058	0.377	2.03 (0.42, 9.93)	0.381	0.49 (0.21, 1.20)	0.120	0.308	0.69 (0.26, 1.89)	0.479
Perceived barrier	0.96 (0.37, 2.48)	0.932	0.981	—	—	0.76 (0.43, 1.34)	0.343	0.637	—	—
Perceived benefit	1.10 (0.46, 2.64)	0.827	0.981	—	—	0.68 (0.40, 1.14)	0.140	0.308	0.72 (0.41, 1.26)	0.249
Self-efficacy	1.11 (1.01, 1.23)	0.029*	0.377	1.08 (0.97, 1.19)	0.163	0.96 (0.90, 1.02)	0.142	0.308	0.93 (0.86, 0.99)	0.044*

**Highly significant, *Significant at 0.05.

a FDR: False Discovery Rate-adjusted *p*-value, calculated using the Benjamini-Hochberg procedure to account for multiple comparisons in the bivariable analyses.

b Variables were selected for inclusion in the multivariable model based on the crude bivariable *p*-value (<0.25), not the FDR-adjusted *p*-value.

## 4 Discussion

This study examined the associations of modifying factors and individual beliefs with smoking behaviors, specifically quit attempts and smoking intensity, among 292 industrial workers in Myanmar, using cross-sectional baseline data from our longitudinal quasi-experimental study (17). The findings provide contextual insights into smoking behaviors in a low-resource occupational setting, independent of any intervention effects.

Compared to similar studies conducted in community or clinical settings, our descriptive analysis revealed low levels of health knowledge (26, 36, 37), perceptions (26, 38, 39), self-efficacy (40), as well as a low prevalence of quit attempts (41). These deficits may be attributed to contextual barriers, such as limited access to health information, time constraints inherent to industrial workers, and a workplace culture that does not prioritize HE. The predominance of physical labor and the social acceptability of tobacco use in such environments likely contribute to a reduced perception of smoking-related risks and diminished motivation to quit. These findings point to a critical need for HE tailored to industrial contexts, where the HBM-based approaches may be more impactful when grounded in real-world behavioral baselines. Although our broader study (17) demonstrated the effectiveness of an HBM-guided intervention combing HE and mobile phone SMS in improving smoking-related health knowledge, perceptions, and self-efficacy, this cross-sectional analysis provides distinct, pre-intervention insights into behavioral predictors. To enhance future intervention design, studies should consider incorporating environmental and social predictors, such as peer influence, media exposure, tobacco accessibility, and workplace culture, into the behavioral framework.

Regarding factors associated with quit attempts, the intention to quit was not significantly associated (*p* = 0.656), which contrasts with theoretical expectations derived from models like the TPB. This may reflect the influence of contextual elements such as economic insecurity, insufficient institutional support, and sociocultural norms, all of which may undermine the translation of intention into action. In this occupational context, individual beliefs may be insufficient drivers of behavior in the absence of enabling conditions. This points to the need for multi-level approaches in future interventions, those that combine individual behavior change strategies with supportive structural changes, including employer-endorsed cessation programs and occupational health policies that discourage smoking.

Notably, none of the HBM perception-based constructs were significantly associated with quit attempts in either the bivariate or multivariable models. This finding contrasts with studies conducted during acute public health threats, such as the COVID-19 pandemic (42, 43), where stronger perceptions of risk prompted more cessation efforts. In contrast, among industrial workers in a relatively stable setting, perceived severity and susceptibility related to smoking may not seem important enough to trigger immediate behavior change. This suggests that in such settings, interventions must actively cultivate a stronger sense of urgency, using approaches like graphic health warnings, narrative storytelling, testimonials, and integration of risk communication into occupational health programs. Future studies should consider longitudinal designs to better assess how these perception shifts evolve and how they influence long-term quit behavior.

Self-efficacy emerged as a significant factor associated with quit attempts (*p* = 0.029) in the bivariate analysis, consistent with the HBM framework and prior studies (44, 45), which affirm the central role of confidence in behavior change. Notably, the effect size (COR = 1.11, 95% CI: 1.01–1.23) indicates a modest increase in the likelihood of quit attempts per unit increase in self-efficacy, suggesting that even small improvements in self-efficacy may be meaningful. However, in the multivariable model, this association was not statistically significant (*p* = 0.163), indicating that self-efficacy may not be independently associated with quit attempts when broader contextual factors are accounted for. This contrasts with Spek et al.’s (31) validation of SASEQ, and our null finding could be partly explained by the lack of structured workplace cessation programs in the Myanmar context. Even so, the bivariable effect size highlights the potential practical relevance of self-efficacy interventions. Without structural and institutional support, high self-efficacy alone may be insufficient to facilitate quit behavior, particularly in environments that do not actively support smoking cessation. In this regard, workplace-based policies that enable and reinforce cessation, such as smoke-free zones, cessation support groups, and peer champion models, may amplify the effectiveness of individual-level interventions.

With respect to smoking intensity, a different pattern emerged. The borderline significance of intention to quit (COR = 1.48, 95% CI: 0.93–2.36, *p* = 0.09) in the bivariable analysis suggests a potentially meaningful effect size, indicating that motivation could still influence cigarette consumption, even if it does not translate into full cessation attempts. While perception constructs did not show a significant relationship with smoking intensity, this may reflect the normalization of tobacco use in the cultural and social fabric of adult industrial workers, a contrast to findings from a U.S. study (46) among adolescents or populations in settings with more prominent anti-smoking messaging. These differences highlight the need for locally tailored HE that addresses cultural acceptance of smoking and highlights immediate occupational health risks, especially in countries with limited anti-smoking enforcement.

Multivariable analysis showed that both health knowledge (AOR = 0.53, 95% CI: 0.38–0.75, *p* < 0.001) and self-efficacy (AOR = 0.93, 95% CI: 0.86–0.99, *p* = 0.044) were significantly associated with lower smoking intensity. These effect sizes indicate that even modest improvements in health knowledge or self-efficacy are linked with meaningful reductions in smoking, reinforcing their practical and clinical significance. This corroborates evidence from previous studies indicating that greater health knowledge of smoking risks (47) and higher self-efficacy (45) are linked with reduced tobacco consumption. These findings reinforce the value of HE programs that not only provide accurate risk information but also empower individuals to act. Even in the absence of complete cessation, reductions in smoking intensity are meaningful public health outcomes, particularly for populations at high risk for NCDs, highlighting the real-world importance of these interventions. Evaluating the long-term impact of reduced smoking on NCD outcomes should be a priority for future research, aligning with health system planning and resource allocation in LMICs. Furthermore, the successful integration of mobile phone-based interventions like SMS, demonstrated in our broader study (17), offers a promising tool to sustain behavior change, especially in settings with limited face-to-face access.

### 4.1 Limitations and strengths

Our study has limitations. First, we could only identify male industrial workers and recruited participants from only two industries, which may introduce selection bias and limits the generalizability of the findings to female workers and other occupational or demographics groups. Future studies should aim to include women, as well as different geographic areas and diverse occupational sectors, to enhance representativeness. Second, the small proportion of participants with a quit attempt (9.6%, *n* = 28) may have limited power and led to imprecise estimates; thus, result should be interpreted cautiously and viewed as exploratory. The *post hoc* power analysis for quit attempts indicated a power of 0.55, suggesting limited ability to detect true associations; therefor these findings should be interpreted with caution. Third, all data were self-reported, including smoking behaviors, which may be subject to recall and social desirability biases. Future research could incorporate biochemical validation to enhance measurement accuracy. Cultural norms and context may have further influenced responses, and although validated scales were used, their reliability in the Burmese cultural setting is uncertain, highlighting the need for culturally adapted instruments and qualitative validation. Additionally, environmental and social determinants such as workplace tobacco policies, peer norms, or stress-related behaviors were not incorporated, which future studies should include for a more comprehensive understanding. Our study was based on pre-COVID baseline data (2018), which may not reflect all post-pandemic changes in smoking behavior, though the identified determinants remain relevant.

Despite these limitations, our study offers important strengths. It addresses a notable gap in the literature on smoking behaviors among industrial workers in Myanmar. By applying the HBM, the study establishes a theoretical framework that connects cognitive and perceptual constructs to behavior outcomes, facilitating targeted intervention development. This theoretical foundation is essential for designing multi-level cessation interventions that pair individual education with systemic support, such as workplace tobacco control frameworks. Our baseline data serves as a crucial foundation for evaluating the effectiveness of future cessation interventions. Importantly, this analysis uses baseline data as a standalone cross-sectional study, distinct from the intervention outcomes of the longitudinal quasi-experimental study from which the data originates. This approach addresses different research questions and employs unique analytical methods focused on behavioral predictors before any intervention exposure. Thus, the findings provide novel insights that complement, rather than duplicate, previous work. Additionally, our study’s focus on a specific occupational group provides valuable contextual insight and highlights leverage points for workplace-based tobacco control initiatives. These findings can inform public health policies aimed at reducing smoking-related NCD burdens and improving worker well-being in similar settings.

## 5 Conclusion

Our baseline study highlights the intricate interactions between modifying factors and individual beliefs influencing smoking behaviors in industrial workers in Myanmar. While health knowledge and self-efficacy are pivotal in reducing smoking intensity, their effect on quit attempts is limited without broader contextual support. These findings highlight the need for theory-informed interventions that integrate behavioral strategies with structural and environmental support to effectively promote smoking cessation in occupational settings.

## Data Availability

The raw data supporting the conclusions of this article will be made available by the authors, without undue reservation.
