# Supplementary material for: Associated factors of smoking behaviors among industrial workers in Myanmar: the role of modifying factors and individual beliefs, guided by the health belief model
Source: Front Public Health. 2025 Sep 16;13:1655922. doi: 10.3389/fpubh.2025.1655922 (PMC12479501; doi:10.3389/fpubh.2025.1655922)
Supplement: Supplementary file 1 [file Table_1.docx]

**Supplemental Material 1**

Health Belief Model Constructs with Definitions and Item-Level Operationalization

| **Construct** | **Definition** | **Item-Level Operationalization** |
| --- | --- | --- |
| Perceived Susceptibility | An individual’s assessment of their likelihood of developing a disease or experiencing a negative health outcome [18]. | Measured by 11 positively worded items rated on a 5-point Likert scale (strongly disagree to strongly agree):   - I am likely to get lung cancer - I am likely to get coronary heart disease - I am likely to get asthma - I am likely to get emphysema - I am likely to get bronchitis - I am likely to have a stroke - I am likely to have circulation problems - I am likely to wheeze - I am likely to cough - I am likely to get angina - I am likely to be unable to enjoy exercise |
| Perceived Severity | An individual’s recognition of the seriousness of a disease or adverse outcome, including potential consequences if no preventive action is taken [18]. | Measured by 10 positively worded items rated on a 5-point Likert scale (strongly disagree to strongly agree):   - Smoking is severe because it causes lung cancer - Smoking is severe because it causes asthma - Smoking is severe because it causes emphysema - Smoking is severe because it causes coronary heart disease - Smoking is severe because it causes bronchitis - Smoking is severe because it causes stroke - Smoking is severe because it causes circulation problems - Smoking is severe because it causes breathlessness - Smoking is severe because it causes cough - Smoking is severe because it causes angina |
| Perceived Barriers | An individual’s assessment of the difficulties or obstacles that might prevent them from carrying out a recommended health behavior [18]. | Measured by 7 negatively worded items rated on a 5-point Likert scale (strongly disagree to strongly agree):   - Buying cigarettes is not a high cost for me - Workers who do not smoke could be estranged from friends who smoke around - Non-smoking could make it harder to start and hold a conversation with a smoker - Non-smoking could limit my social activities (parties, bars, coffee shops, etc.) - Non-smoking could lead to loss (current smokers) or lack of one's identity - Smoking is a critical way to handle the stress from work - Smoking is an effective way to handle one's mood when getting upset |
| Perceived Benefits | An individual’s belief in the advantages or effectiveness of taking specific actions to reduce the risk or impact of a health condition [18]. | Measured by 5 positively worded items rated on a 5-point Likert scale (strongly disagree to strongly agree):   - Non-smoking can save me much money for buying foods, doing other things I want - Non-smoking can help me keep out of contracting some serious diseases like lung cancer, asthma, coronary heart disease, etc. - Non-smoking can help me cast off some physical discomfort (cough, throat, bronchitis, and so on) - Non-smoking can help me keep physical stamina for enjoying exercises I like - Non-smoking can make me attractive to those who dislike the smell of smoking |
| Self-Efficacy | An individual’s confidence in their ability to successfully carry out a specific behavior, which influences their likelihood of engaging in that behavior [18]. | Assessed using 6 items from the Smoking Abstinence Self-Efficacy Questionnaire (SASEQ), with a question “Are you confident that you will not smoke?”, rated on a 5-point scale from "Certainly Not" (score 0) to "Certainly" (score 4):   - When you feel agitated or tense - When you are (very) angry - When you are in a café, at a party, or paying a visit - When you feel (very) sad - When someone offers you a cigarette of your own brand - When you see someone enjoy smoking |
| Cues to action | It refers to internal or external triggers, such as environmental influences or personal experiences, that prompt individuals to initiate recommended health behaviors [18]. | It was not assessed as a baseline construct in this study because they were operationalized as part of the intervention (i.e., health education sessions and mobile phone short message service reminders) delivered during the quasi-experimental phase [17]. |
